# Quality Health Care in the European Union Thanks to Competition Law

**DOI:** 10.3390/ijerph7010001

**Published:** 2009-12-24

**Authors:** Diego Fornaciari

**Affiliations:** KU Leuven, Centre for Biomedical Ethics and Law, Kapucijnenvoer 35, 3000 Leuven, Belgium; E-Mail: Diego.Fornaciari@med.kuleuven.be; Tel.: +32 16337030; Fax: +32 16336952

**Keywords:** antitrust, competition law, quality of health care, health policy

## Abstract

There are many biases concerning the application of competition law in health care. Quality concerns can however be integrated into competition law analysis. The aim of this paper is to identify the links between the application of competition law in the European Union and the right to quality health care and to point out the problems that arise when integrating quality concerns in competition law analysis. Guidelines must be issued and competition authorities must work together with institutions that have expertise in the field of health care quality measurement in order to integrate these dimensions in competition practice.

## 1. Introduction

Concerning the question of the application of competition law to the European health care sector, opinions are divided. Lawyers argue that the conditions for the application of competition rules to health care players are generally met. Policymakers and health care institution managers are sometimes sceptical about the idea of applying competition law to health care. They often argue that health care is very different from other sectors in our economy. Hospitals, for example, do not strive for profit and they perform a service of general economic interest, therefore, they should not be submitted to the rules concerning a free market. Nevertheless, the application of competition law in health care is not *a priori* harmful, as competition law aims at protecting the patient. Moreover, the application of competition law does not necessarily reflect a choice for more competition and deregulation. The aim of competition law is indeed to protect the consumer and, in the health care sector, the patient, from conduct that is anticompetitive, deceptive or unfair [1]. This paper will analyze the links between competition law and the right to quality health care of the patient. The right to quality health care, which is legally recognized in several countries in hospital laws and patient rights, gives e.g., the patient, in his relationship with the medical professional (and public authorities), the right to quality care that satisfies his needs, with respect of his human dignity and his right to self determination and without any discrimination on whatever ground (this is the right as provided in the Belgian patient rights law). To assert what can be regarded as quality care, judges take into consideration e.g., the standards, guidelines and other documents provided by scientific professional organizations. The findings below will show that quality of care is considered in different aspects of the competition law analysis.

## 2. Results and Discussion

### 2.1. Competition Law Is Applicable to Health Care Players

Competition law can be divided into several policy areas. It prohibits agreements and practices which restrict free trading and competition between business entities and bans abusive behavior by a firm or institution dominating a market, or anticompetitive practices that tend to lead to such a dominant position. Concentrations (mergers and acquisitions that exceed certain thresholds) are submitted for approval by the qualified competition authorities. The competition authority will then investigate if the merger or acquisition is likely to lead to a dominant position, a restriction of trade, *etc.* Under European competition law, direct and indirect aid given by member states is controlled by article 87 EC-treaty [2].

The question is if health care players can be considered as undertakings, in order for them to fall within the scope of competition law. Based on an examination of case law and literature this question can be answered positively. Hospitals, health professionals, health insurers (in as far as their actions do not involve the compulsory health insurance), pharmaceutical firms, pharmacists, *etc.* are undertakings because they perform economic activities [3]. Thus, health care players fall generally within the scope of competition rules, even in countries where there is little competition among health care players because of, e.g., extensive regulation. Therefore, when hospitals enter into agreement with other hospitals or with a pharmaceutical firm, they will have to comply with competition law.

### 2.2. Barriers to Effective Competition in the Health Care Market

#### 2.2.1. Competition law presumptions

The core assumption of competition law is that competitive markets will lead to an efficient allocation of goods and services, to the lowest prices, the highest quality, *etc.* Following this assumption, as health care players enter into competition, the patient should receive high quality care. According to Hyman [4] however, the health care sector has different characteristics that pose barriers to improving quality. Quality considerations play an important role in a patient’s selection of a health care provider. As patients do not have ready access to information on quality, they will not be able to exercise their preferences along the dimensions of health care quality that are important to them. This informational barrier prevents competition to have the same results in the health care market as in other markets with more quality transparency. There can be no competition based on quality when the patient lacks sufficient information.

Therefore, various initiatives have to be undertaken to make quality information more accessible for the patient. In the Netherlands, transparency is already considered a very important issue in health care and several authorities are appointed to take steps to improve quality transparency in health care, *i.e.*, the National Health Care Authority (hereinafter NZa) and the Inspection for Health Care (hereinafter IGZ). The NZa is qualified for transparency in health care in general (*i.e.*, not only quality transparency) [5] and the IGZ is qualified for quality transparency. The NZa can publish information itself, it can retrieve information from health care players, it can set rules concerning the publication of information by health care players, *etc.* Such initiatives by an independent health care authority, or even maybe the competition authority, can be of great importance for quality transparency. However, it is necessary for these authorities to work together with other institutions that are specialized in quality measurement (such as the IGZ), to make sure that the information is correct and reliable for the patient.

#### 2.2.2. How does competition law improve quality?

The application of competition law improves the quality of care by protecting the patient against unfair and anticompetitive practices such as abuse of dominant position, distribution agreements, *etc.* On the other hand, quality concerns can also be introduced in the competition law analysis (2.3). Some practices by medical professionals will be aimed at improving the quality of care, even if they restrict competition. In some cases, competition law will allow these practices if they provide qualitative efficiencies to the benefit of the patient. In the United States, where health care players have a lot more possibilities to enter into competition, as opposed to European countries, competition law has opened the door to alternative practitioners and forms of practice and enhanced quality by maximizing choice in the marketplace [6].

#### 2.2.3. The results from empirical research

The results of empirical research on the relationship between competition and health care quality are not consistent [7–9]. In markets where prices are regulated by the government, e.g., Kessler and McClellan [10] and Tay [11] found a positive effect of competition on the quality of care. Gowrinsankaran and Town [12] on the other hand, found a negative effect. In markets where prices are not regulated but set by the companies, the results are even more variable. As it is not the scope of this article, I will not elaborate furthermore on the impact of competition on health care quality. Gaynor, whose article gives an overview of literature on this subject, believes that the next step in research must be to determine the factors that influence the impact of competition on the quality of care [7].

### 2.3. Quality in the Competition Law Analysis

#### 2.3.1. Introduction

The question if a practice, agreement or decision by an (association of) undertaking(s) is anticompetitive is subject to a competition law analysis. This analysis is mainly based on price considerations. Quality as a competitive dimension is often ignored [13]. The main reason is that quality as a concept cannot be defined easily [14]. The concept consists of multiple dimensions and dependent on the point of view a different interpretation can be given [15]. Because quality is difficult to define and measure, it is also difficult to assert if a practice has a positive or negative impact on the quality of care. Unfortunately, competition authorities do not have much expertise in the field of health care quality [16]. Nevertheless, a review of European and national regulation, case law and literature shows that quality can sometimes be integrated into the competition law analysis, especially the “efficiency”-dimension [17].

#### 2.3.2. Efficiency as a dimension of quality in competition law

Agreements that restrict competition can, at the same time, provide efficiency gains that contribute to improving competition. Efficiencies may create additional value by lowering the cost of producing an output, improving the quality of the product or creating a new product. When the pro-competitive effects of an agreement outweigh its anti-competitive effects the agreement is on balance pro-competitive and compatible with the objectives of the Community competition rules [18]. Hospitals can agree to collectively provide some hospital services to prevent overlap, to improve the quality of the hospital service and to attend to the needs of the population. This type of agreement can be anti-competitive, but if the pro-competitive effects, provided by the improvement of quality and the efficient allocation of services, outweigh the anti-competitive, it can fall under the exemption. This exemption is provided under European competition law in article 81 (3) EC-Treaty. For the application of this article four conditions have to be fulfilled. The agreement: (1) must contribute to improving the production or distribution of goods or contribute to promoting technical or economic progress; (2) consumers must receive a fair share of the resulting benefits; (3) the restrictions must be indispensable to the attainment of these objectives and (4), the agreement must not afford the parties the possibility of eliminating competition in respect of a substantial part of the products in question.

For health care it is of great importance that cost efficiencies, as well as qualitative efficiencies can be considered when applying article 81 (3) EC-Treaty. In its communication of April 27, 2004, the European Commission expressively states that in some cases quality improvements and other qualitative efficiencies can be the most important efficiency gain provided by an agreement. Unfortunately, in practice, qualitative efficiencies are rarely considered in the competition law analysis. In some cases a quality decrease is considered as an anti-competitive disadvantage. In the case *Amicon Verzekeraar Vrijgevestigd Fysiotherapeut*, the Dutch Competition Authority (hereinafter NMa) considered that pricing agreements for the purchase of physiotherapeutic care do not only eliminate price competition, but also discourage physiotherapists to compete on quality of services.

A merger can also provide efficiency gains that outweigh the negative effects of the merger on competition [19]. The relevant benchmark in assessing efficiency claims is that consumers will not be worse off as a result of the merger. For that purpose, efficiencies should be substantial and timely, and should, in principle, benefit consumers in those relevant markets where it is otherwise likely that competition concerns would occur. Efficiencies are relevant to the competitive assessment when they are a direct consequence of the notified merger and cannot be achieved to a similar extent by less anticompetitive alternatives. In these circumstances, the efficiencies are deemed to be caused by the merger and thus, merger-specific. As in most circumstances it will be able to obtain the same qualitative efficiencies by other agreements, less radical than a concentration, this requirement will rarely be fulfilled.

Recently, in the case *Ziekenhuis Walcheren-Oosterscheldeziekenhuizen* on March 25, 2009, the NMa allowed a concentration of hospitals based on an efficiency defense. According to the hospitals, the concentration was necessary because in time it would be harder for them to provide even basic care and the risk that just one of the hospitals could survive was deemed realistic. In order for the hospitals to fulfill their qualitative goals and to guarantee the continuity of care, a concentration was necessary. According to the hospitals the efficiency gains would outweigh the anti-competitive consequences. For its assessment of the quality improvements, the NMa worked together with the IGZ, which is specialized in quality measurement. The IGZ gave an advisory opinion in this case, finding that the merger would indeed provide qualitative efficiencies, but that these efficiencies were not sufficiently verifiable. As a result, the parties suggested some remedies for the merger to take place, such as the obligation to report to the NMa some time after the merger took place, to verify that the qualitative efficiencies were accomplished. Finally, the NMa allowed the merger on these grounds. It was the first time qualitative efficiencies had been evaluated for a hospital merger. The decision of the NMa (together with other decisions in hospital merger cases) was criticized by competition law scholars. Concerns rose especially regarding the magnitude of the hospitals (and their market dominance) [20]. An appeal decision in this case is awaited.

#### 2.3.3. Services of general interest, quality of care and regulation regarding state aid

In European competition law, quality is indirectly an important topic in the relationship between health care players and the government concerning services of general economic interest. In accordance with article 86 (2) EC-Treaty, undertakings entrusted with the operation of services of general economic interest or having the character of a revenue-maximizing monopoly are subject to the competition rules, insofar as the application of such rules does not obstruct the performance, in law or in fact, of the particular tasks assigned to them. The service of general interest is necessary in order to ensure continuity and quality of care. When the government entrusts health care players with the operation of a service of general interest health care quality is an important aspect. The government often imposes obligations for these services such as continuity (the obligation to provide the service regardless of the fact whether or not there is sufficient demand for this service), minimal requirements regarding quality, frequency of the provided service, *etc.* Because of the exemption of article 86 (2) EC-Treaty, anti-competitive practices can be allowed when they are necessary to perform the tasks of general economic interest, e.g., because they are necessary to provide a qualitative service [21].

Article 86 (2) EC-Treaty is also of importance for state aid regulation (article 87 EC – Treaty). In the *Altmark Trans* case the European Court of justice has set the conditions to accept compensations provided for services of general economic interest. The recipient undertaking must actually have public service obligations to discharge and those obligations must be clearly defined. The parameters on the basis of which the compensation is calculated must be established both in advance and in an objective and transparent manner. The compensation cannot exceed what is necessary to cover all or part of the costs incurred in the discharge of the public service obligations, taking into account the relevant receipts and a reasonable profit [22]. Procedurally, this means that EU member states need not notify such compensation to the Commission. When these conditions are met, compensations provided for services of general economic interest, even if they distort or threaten to distort competition by favoring certain undertakings or the production of certain goods, will be compatible with the common market.

Whether or not health care players are entrusted with services of general economic interest is difficult to say, and needs a case by case approach. The act of entrustment may be by way of legislative measures or regulation. An undertaking may also be entrusted through the grant of a concession or license governed by public law. According to the European Commission [23], e.g., the Dutch health insurers are entrusted with a service of general economic interest when they provide the compulsory health insurance. Therefore, they fall within the scope of article 86 (2) EC-Treaty.

### 2.4. Discussion

Although quality concerns can be integrated into the competition law analysis, in practice they rarely are. Judges don’t have the expertise to verify quality improvements or decreases caused by agreements or practices by undertakings. Therefore guidelines must be issued in order for courts to be able to integrate the concept of quality in their analysis. These guidelines are particularly necessary for the application of qualitative efficiencies under the exemption of article 81 (3) EC-Treaty and in concentration control. To issue these guidelines, competition authorities have to work together with institutions, specialized in quality of care measurement, as they do have the expertise to adequately analyze quality of care issues. The example was given of the Netherlands, where the NMa works together with the NZa and the IGZ. They are authorized to advise in certain cases and they work together with the NMa in publishing policy papers. Guidelines are also necessary involving services of general economic interest. It is not clear if or when health care players are entrusted with the operation of services of general economic interest. To provide certainty on the requirements the European authorities need to issue clear guidelines.

## 4. Conclusions

Health care players fall generally within the scope of competition rules, even in countries where there is little competition among health care players because of e.g., extensive regulation. The application of competition rules on the health care market does however not imply a choice for more competition. Competition law aims at protecting the patient and competitors against conduct that is anticompetitive, deceptive or unfair.

Quality as a competitive dimension is often ignored by courts. The main reason is that quality as a concept can not easily be defined. There are however links between competition law and quality health care. Efficiency is a dimension of quality that can be integrated in the competition law analysis. Indirectly, quality is also an important topic in the relationship between health care players and the government concerning services of general economic interest. It is not clear if or when health care players are entrusted with the operation of services of general economic interest.
